# The association between occupational loading and spine degeneration on imaging – a systematic review and meta-analysis

**DOI:** 10.1186/s12891-019-2835-2

**Published:** 2019-10-27

**Authors:** Luciana G. Macedo, Michele C. Battié

**Affiliations:** 1 School of Rehabilitation Science (Physiotherapy), Faculty of Health Sciences, McMaster University, 1400 Main St. W. Room 441, IAHS, Hamilton, ON L8S 1C7 Canada; 2 Western University, London, Ontario Canada

**Keywords:** Occupational load, Spine degeneration, Disc degeneration, Disc height, Imaging, Magnetic resonance imaging, X-ray

## Abstract

**Background:**

There are inconsistencies in findings regarding the relationship of occupational loading with spinal degeneration or structural damage. Thus, a systematic review was conducted to determine the current state of knowledge on the association of occupational loading and spine degeneration on imaging.

**Methods:**

We performed electronic searches on MEDLINE, CINAHL and EMBASE. We included cross-sectional, case control and cohort studies evaluating occupational loading as the exposure and lumbar spine structural findings on imaging as the outcomes. When possible, results were pooled.

**Results:**

Seventeen studies were included in the review. Ten studies evaluated the association of occupational loading with disc degeneration (signal intensity), four of which were pooled into a meta-analysis. Of the 10 studies, only two did not identify a relationship between occupation loading and disc degeneration. A meta-analysis including four of the studies demonstrated an association between higher loading and degeneration for all spinal levels, with odds ratios between 1.6 and 3.3. Seven studies evaluated disc height narrowing and seven evaluate disc bulge, with six and five identifying an association of loading and with imaging findings respectively. Three studies evaluated modic changes and one identified and association with occupational load.

**Conclusions:**

There was moderate evidence suggesting a modest association between occupational loading and disc degeneration (signal intensity), and low-quality evidence of an association between occupational loading and disc narrowing and bulging.

## Background

The cumulative or repetitive injury model was once a dominant paradigm of spine degeneration [1]. Thus, heavy occupational physical loading activities have long been suspected of increasing spine degeneration. However, inconsistencies between study findings, with some supporting this association [2, 3] and other not [4, 5], have led to controversy and uncertainty about the relationship between physical loading and lumbar spine degeneration. Furthermore, recent studies suggest that the structures of the spinal column, including the intervertebral discs, adapt and may even benefit from greater routine physical loading [1].

Controversy still exists between the relationship of occupational load and low back pain [6]. However, given the subjective nature of pain evaluation and the high prevalence of back pain in general, studies depicting the association between pain and occupation load always have large room for bias. The use of objective measures of spine degeneration to evaluate the impact of occupational load on the spine can provide a solution to better understanding this relationship. The evaluation of spine degeneration on imaging is both a reliable and objective measure to evaluate the effects of repetitive load on the spine, which in turn may mediate the occurrence of back pain in this population. Although spine degeneration on imaging is not synonym of back pain, spine degeneration on imaging have been found to be associated with an increased risk for low back pain [7] and increased risk of recurrent episodes [8].

Given the inconsistencies in the literature about the association of occupational load and spine degeneration, the objective of this study was to systematically review the literature on the association of occupational loading and spine degeneration observed on imaging. Occupational loading was described as loading conditions occurring during occupational activities, such as lifting and manual handling or comparisons between specific occupations.

## Methods

A protocol for the study was developed a priori following the PRISMA guidelines and Cochrane Handbook.

### Data sources and searches

A computerised electronic search was performed to identify relevant articles. The search was conducted on MEDLINE (1946 to May 2019), CINAHL (1982 to May 2019) and EMBASE (1988 to May 2019). Key words included in our search were related to 3 domains: imaging (i.e. x-ray, radiograph), imaging findings (i.e. disc degeneration, disc height) and load (i.e. manual handling, occupational load). Subject subheadings and word truncations specific for each database were used. There was no language restriction. See Additional file 1 for search strategy section.

Two reviewers screened search results (titles and abstracts) for potentially eligible studies. A third independent reviewer resolved any disagreement for inclusion of trials. Authors were contacted if more information about the trial was needed to allow inclusion of the study.

We also performed a search on the reference lists of the included studies and a search on ISI Web of Sicence (May 2019) for papers that cited the included studies.

### Study selection

Cross-sectional, case-control and cohort studies evaluating occupational loading as the exposure were eligible for inclusion. All studies that evaluated professional athletes and whole body vibration as a form of exposure were included on a separate review. In addition, the study had to evaluate the relationship of loading with lumbar spine structural findings evaluated on diagnostic imaging. Studies that used back pain as an outcome measure were not included. Studies that included patients with pre-existing conditions, such as disc herniation, were excluded from the review as they are more likely to have positive findings on imaging and may provide biased estimates for the relationship under investigation. Two reviewers screened the full text of potentially eligible studies and decided on inclusion. A third independent reviewer resolved any disagreement for inclusion of studies. The reviewers followed a research protocol developed prior to the beginning of the review, which included a checklist of inclusion criteria.

### Data extraction and quality assessment

The methodological quality of the trials was assessed using the Newcastle Ottawa Quality Assessment scale [9, 10] for case-control and cohort studies. The maximum value of the scale is 9 (high quality) and the minimum value is 0 (lowest quality). The quality was assessed by independent raters and disagreements were resolved by a third rater. Methodological quality was not an inclusion criterion but was taken into consideration when making conclusions.

Two independent reviewers (LM and research assistants) extracted data from the included studies using a standardized data extraction form. Important characteristics of each study were extracted, such as type of loading, study design, type of imaging, patient population, affiliation of the authors, funding source, and study conclusions. We also extracted the type of outcomes used, and for continuous outcome measurements we extracted mean scores, standard deviations and sample size, and for dichotomous and ordinal outcomes, sample size and number of events per group.

### Data synthesis and analysis

Results were pooled when trials were considered sufficiently homogenous with respect to participant characteristics, exposure and outcomes. I^2^ was calculated using RevMan 5 to assess statistical heterogeneity. A random effects model was used to pool all available outcomes. I^2^ was calculated to evaluate statistical heterogeneity of pooled outcomes [11]. When adequate data were presented from the original study, mean differences and standard deviations for continuous outcomes and odds ratios for dichotomous outcomes were calculated. When such information was not available, the information presented in each study was used for interpretation of the results.

The GRADE approach for grading the level of the evidence available was used to summarize the conclusion of this review [11]. Depending on the number and quality of the studies included in the review, the evidence was classified into high, moderate, low or very low quality evidence.

## Results

### Study selection

The electronic database search resulted in a total of 5363 articles after removing duplicates. Of these, 137 were selected as potentially eligible based on their title and abstract.

**Fig. 1:**
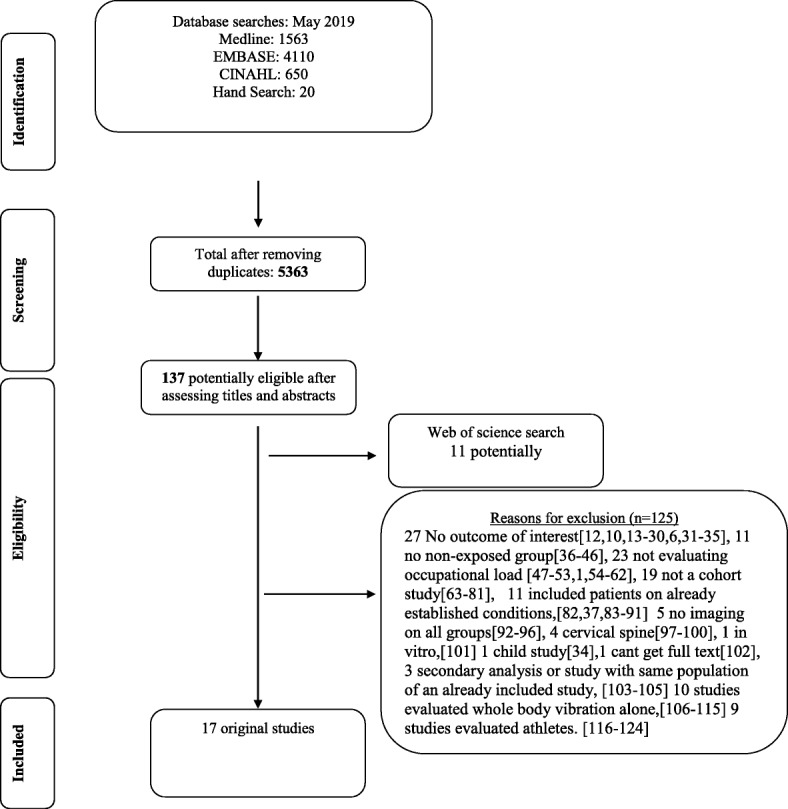
Flow chart of occupational load systematic review inclusion

After full title screening a total of 16 studies were included in the review. An additional ISI web of science search showed 11 more potentially eligible studies, from which 1 was included in the review. Therefore, the final number of included studies was 17. (Flowchart_ Fig. 1).

125 Exclusion for graph only * 27 No outcome of interest [6, 10, 12–30, 31–35], 11 no non-exposed group [36–46], 23 not evaluating occupational load [1, 47–53, 54–62], 19 not a cohort study [63–81], 11 included patients on already established conditions, [37, 82–91] 5 no imaging on all groups [92–96], 4 cervical spine [97–100], 1 in vitro, [101] 1 child study [34], 1 cant get full text [102], 3 secondary analysis or study with same population of an already included study, [103–105] 10 studies evaluated whole body vibration alone, [106–115] 9 studies evaluated athletes [116–124].

### Study characteristics

There were 16 original studies evaluating occupational load [2–5, 125–136] including 1 follow-up study [137]. Ten studies evaluated specific job requirements such as occupational lifting or job load summary score [2, 4, 127–130, 132, 133, 135–137] and six compared different types of occupation with different occupational requirements [3, 5, 125, 126, 131, 134]. See Table 1 for study characteristics.

**Table 1:** Study characteristics

Author	Methodological Quality	Imaging	Study Design	Participant information
Arevalo et al. 2014	5	MRI	Retrospective cohort study	*N* = 652; 326 patients with established diagnosis of lumbar disc herniation on MRI and 326 patients without herniation. Age range was assessed but not reported.
Battie et al. 1995	6	MRI	Cross sectional	Monozygotic twin pairs from the Finnish Twin Cohort selected based on loading discordances. (*n* = 230 twins or 115 pairs) Age range from 35 to 69 years.
Biering-Sorense et al. 1985	4	x-ray	Longitudinal cohort study	666 participants taking part in a population study of 60-yerar-old inhabitants from the area around Glostrip Hospital in the suburbs of Copenhagen. All participants were either 50 or 60 years of age.
Brinckmann et al. 1998	5	x-ray	Retrospective cohort study	355 subjects with long term exposure to heavy physical load (from different professions) and 737 healthy controls compiled from healthy unexposed subjects. Participants were between 17 to 57 years old.
Elfering et al. 2002	3	MRI	longitudinal cohort study	46 asymptomatic patients from a group of 2000 trauma patients presenting to the university trauma clinic with minor extremity injuries with complete recovery. Participants were between 20 and 50 years of age.
Frymoyer et al. 1984	3	x-ray	Cross sectional (retrospective)	321 random patients from a sample of 1221 from a previous study. Only 285 were included in the occupation vs imaging study due to reasons such as imaging quality Participants were between 18 an d55 years of age.
Han et al. 2017	6	MRI and x-ray	Cross sectional	210 patients with low back pain attending the hospital. All underwent imaging evaluation and responded to questionnaires about workload. Age ranged from 40 to 60 years.
Hangai et al. 2008	4	MRI	cross sectional	Recruited over 50 year old to participate in a health promotion program though newspapers in Japan. Those with imaging were recruitment. *N* = 270 (1350 discs) All participants were between 51 and 86 years.
Hartwig et al. 1997	4	MRI	Cross sectional	Recruited 142 participants from 35 to 50 years old that were either nurse (*n* = 54), construction workers (*n* = 51) or controls (*n* = 37). Unclear how controls were selected All patients were between 35 and 50 years old.
Hung et al. 2014	6	MRI	Cross sectional	553 workers that carry heavy loads (fruit market workers) versus walk in clinic patients (most commonly diagnosis was common cold). After that all participants were assessed using questionnaires and functional assessment for the amount of lifting load and then categorized into either low, intermediate, or high lifting loads. Participants were between 20 and 65 years old.
Luoma et al. 1998	8	MRI	cross sectional	Patients were extracted from a cohort participating in a study evaluating occupational effects of LBP. (*n* = 164, 53 drivers, 51 carpenters and 60 office workers). Participants were between 40 and 45 years old.
Munoz-Gomez et al. 1980	5	x-ray	Cross sectional	Workers from an industry All participants were between 19 to 63 years of age.
Riihimaki et al. 1990	6	x-ray	cross sectional	*N* = 417 Male workers. 216 concrete reinforcement workers of an specific area were included and 201 house painters from a local union. Painters were matched with concrete workers based on a 5-year strata. Participants were between 25 to 54 years of age.
Savage et al. 1997	4	MRI	cross sectional (only 60% participated in the longitudinal cohort)	Volunteers from different occupations. *N* = 149 (24 ambulance men, 16 hospital porters, 40 car production workers, 12 brewery drayman and 57 office workers). All participants were between 20 and 58 years of age and were divided in two groups 20–30 and 31 to 58 years.
Schenk et al. 2006	7	MRI	Cross sectional - case control	*N* = 109 staff of local hospital that worked at least 20 h per week. (57 nurses and 52 administration workers) All participants were between 45 and 62 years old.
Videman et al. 2006	7	MRI	longitudinal cohort study	Monozygotic twin pairs from the Finnish Twin Cohort selected based on smoking, exercise or occupational loading discordance. (*n* = 140 twins or 70 pairs). All participants were between 35 to 69 years of age.
Videman et al. 2007	7	MRI	restrospective cohort study	*N* = 600 patients from the Finnish Twin Cohort. 474 were included in the model for disc signal and 513 in the model for disc height (inclusion was dependent on availability or imaging and occupational data) All participants were between 35 to 70 years of age.

Methodological quality was assessed using the Newcastle Ottowa Assessment Scale for case control studies or cohort studies accordingly. Scores are given in starts with a maximum (higher quality) of 9 starts

### Methodological quality

The methodological quality demonstrated an overall moderate level of quality, with a minimum of 3, a maximum score of 8, and a median and interquartile range of 5 and 3. The items of the methodological quality scale that were not present in most studies were control of potentially confounding factors and reporting of the response rate of each group.

Three studies included the same population from the Finnish Twin Spine study, but answered different questions related to different outcomes. These studies represent the strongest form of evidence given that controls are identical twins, minimizing possible confounding and familial aggregation [127, 128].

### Outcomes

Ten studies evaluated the impact of loading on disc degeneration, generally assessed through disc signal intensity, representing disc desiccation [2–5, 126–129, 133, 134, 137]. The primary method of evaluating disc degeneration is through observing disc signal intensity on imaging. Disc degeneration is often associated with a whiter less translucent appearance of the disc [126]. Of these 10 studies only two did not find significant differences between groups [3, 129] and one study found more degeneration in those with less load [127]. In one study we were not able to assess whether statistical differences existed [134]. Seven studies identified some significant difference between loading groups with more load being associated with more degeneration, although we were not able to pool the results given the differences in types of loads and outcomes measured [2, 4, 5, 126–128, 133]. For all comparisons odds ratios when calculated varied between 1.89 to 3.7. A summary of the findings is presented in Table 2. One additional study looked at an overall measure of degeneration that included a combination of factors, and found that occupational loading was associated with the overall degeneration measurements [130].

**Table 2:** Exposure and results of each study included in the review that evaluated occupational load

Study	Type of loading or exposure	Outcomes	Study results and RevMan analysis
Disc degeneration			
Battie et al. 1995	job code (1–4), total occupational lifting (day), mean time working twisted/bent, mean time sitting at work, occupation driving (hrs lifetime)	Disc degeneration (signal intensity)	There was an association between job code (0–4), occupational lifting and occupational sitting with disc degeneration. Greater occupational loading/lifting was associated with greater disc degeneration but associations were small (r = 0.18–0.31) Those with sitting had less disc degeneration.
Biering-Sorensen et al. 1985	work is sedentary, light manual or heavy manual; worker undertakes heavy manual work, amount of physical activity at work	Relative disc degeneration (method was unclear) (for each level from L1 to S1)	RevMan: there was statistically significantly greater for L4 disc degeneration in daily manual workers compared to seldom manual workers (OR = 2.27; 95% CI 1.21 to 4.25), but no difference in disc degeneration at L5 (OR = 1.21; 95% CI 0.44 to 3.36) for physical activity at work. All other comparisons for disc degeneration were not significant but data was not presented.
Elfering et al. 2002	Frequent lifting or carrying heavy objects, forward bending, vibration, sedentary activity, working night shifts	Disc degeneration (1–5 Pearce score) (summary score for all levels together)	The association of working night shifts and disc degeneration did not reach statistical significance (OR = 9.58 95% CI 1.00 to 91.62)
Hangai et al. 2008	Lifting more than 10 kg for more than one third of the working hours.	Disc degeneration (signal intensity with modified Pirfmann’s classification) (for each level from L1 to S1)	RevMan: Occupational lifting was not significantly associated with degeneration at any of the levels. L1 L2 (OR = 3.16 95% CI 0.37 to 26.75), L2 L3 (OR = 1.92 95% CI 0.20 to 18.61), L3 L4 (OR = 1.34 95% CI 0.05 to 38.91), L4 L5 (OR = 2.23 95% CI 0.21 to 23.84) and L5S1 (OR = 1.48 95% CI 0.09 to 23.88)
Hartwig et al. 1997	Nurse, construction workers and controls	Unclear disc degeneration measure assessed as mono, bi, tri or multi-segmental.	Not enough data to calculate an odds ratio. Results suggest that 17% of patients with high workload had mono-segmental degeneration as opposed to 29% of those with no workload, suggesting that those with more workload had degeneration at more levels.
Hung et al. 2014	Workers that carry heavy loads divided into low, intermediate and moderate lifting loads.	Disc dehydration (T2-weigthed signal intensity loss	There was a statistically significant difference in disc degeneration (dehydration) between lifting loads for L1 L2 (OR = 2.4 95% CI 1.4 to 4.0), L2 L3 (OR = 3.3 95% CI 1.3 to 3.2), L3 L4 (OR = 3.7 95% CI 2.4 to 3.5), L4 L5 (OR = 4.9 95% CI 3.0 to 8.0) and L5S1 (OR = 3.6 95% CI 2.3 to 5.7) when comparing the high load to the low load groups. There was also a significant difference between the intermediate and low load groups for L2 L3, L3 L4, L4 L5 and L5S1.
Luoma et al. 1998	Drivers, carpenters and office workers	Disc signal intensity (L2 L3-L5S1)	RevMan: There were no differences between groups. L2 L3 (OR = 0.55 95% CI 0.16 to 1.96), L3 L4 (OR = 1.50 95% CI 0.66 to 3.42), L4 L5 (OR = 2.04 95% CI 1.35 to 3.08) and L5S1 (OR = 1.30 95% CI 0.70 to 2.43)
Savage et al. 1997	ambulance workers, hospital porters, car production workers, brewery drayman and office workers	Disc degeneration (signal intensity) (all levels together)	RevMan: There was no difference between groups in relation to disc degeneration. Car production vs office workers (OR = 1.00 95% CI 0.34 to 2.94); hospital porters vs office workers (OR = 1.63 95% CI 0.45 to 5.91
Schenk et al. 2006	nurses and office workers	Disc degeneration (signal intensity) (1–5)	RevMan: Disc degeneration was different between occupational groups for grade 2 with more degeneration in nurses (OR: 1.89; 95% CI 1.34 to 2.66, *n* = 544 all levels) and grade 4 with more degeneration in office workers (OR = 0.50 95% CI 0.29 to 0.86). There was no difference for grade 3 (OR = 1.14 95% CI 0.80 to 1.64) and grade 5 (OR = 0.75 95% CI 0.37 to 1.52),
Videman et al. 2007	Job code (1–4) History of lifting at work (1000 kg)	Disc signal intensity (L1-S1)	There was a significant association between history of lifting at work and signal intensity in the opposite direction (better signal with more load (Regression coefficient 0.001, *p* = 0.002), there was no association of occupational loading scoring and disc degeneration.
Disc height			
Battie et al. 1995	job code (1–4), total occupational lifting (day), mean time working twisted/bent, mean time sitting at work, occupation driving (hr lifetime)	Disc height	There was an association between job code (0–4), occupational lifting and occupational sitting with disc height but the association was not strong r = −0.22)
Biering-Sorensen et al. 1985	work is sedentary, light manual or heavy manual; worker undertakes heavy manual work, amount of physical activity at work	Disc height (for each level from L1 to S1)	There were no significant differences for disc height
Brinckmann et al. 1998	Different occupations such as mining, steelworkers and normative data of unexposed individuals	Disc height (for each level from T12 to S1)	Occupational loading was associated with a smaller disc height at a few spinal levels, particularly in those working in underground mines.
Hung et al. 2014	Workers that carry heavy loads divided into low, intermediate and moderate lifting loads.	Disc height narrowing (Farfan method. L4 L5 and L5S1	RevMan: There were significant differences between groups for disc height narrowing at L5S1 (OR = 5.8 (2.7 to 13.6)).
Riihimaki et al. 1990	Concrete reinforcement workers and house painters	Disc space narrowing (0–5 for each level)	RevMan: Concrete workers had greater disc height narrowing overall (OR = 2.19; 95% CI 1.34 to 3.58), L3 L4 (OR = 5.34; 95% CI 1.17 to 24.39) and L4 L5 (OR = 2.54; 95% CI 1.26 to 5.11) than painters. There was no difference at L1 L2 (OR = 2.84 95% CI 0.57 to 14.25), L2 L3 (OR = 0.93 95% CI 0.13 to 6.66) and L5S1 (OR = 1.35 95% CI 0.73 to 2.48).
Videman et al. 2006	Job code (1–4), occupational driving, maximum weight lifted at work (kg)	Disc height narrowing	There was an association between occupational lifting and changes in degeneration over 5 years. (0.1 points/disc decrease in disc height = 0.021)
Videman et al. 2007	Job code (1–4)	Disc height T12-S1)	There was an association between lifetime occupational loading score and disc height (regression coefficient 0.038, *p* = 0.004) and no association between history of lifting at work and disc height.
Overall measure of degeneration			
Munoz-Gomez et al. 1980	Work load calculated as above or below the general average	Degeneration (osteophytes, disc bulge and costotransversal arthrosis)	RevMan: Those with occupational load greater than the average had greater degeneration (OR = 1.63; 95% CI 1.03 to 2.57).
Disc Bulge or herniation			
Arevalo et al. 2014	Heavy physical work activities	Disc herniation	There was an association between heavy physical work and disc herniation (OR = 2.0; 95%CI 1.42 to 2.76)
Battie et al. 1995	job code (1–4), total occupational lifting (day), mean time working twisted/bent, mean time sitting at work, occupation driving (hr lifetime)	Disc bulging (summary score for upper T12-L4 and lower lumbar spine L4-S1)	There was no association between occupational loading and disc bulging
Hung et al. 2014	Workers that carry heavy loads divided into low, intermediate and moderate lifting loads.	disc bulging, L4 L5 and L5S1	There was a statistically significant difference in disc bulging between lifting categories for L2 L3 (OR = 3.8 (2.3 to 6.3)), L3 L4 (OR = 3.6(2.4 to 5.6)), L4 L5 (OR = 3.1 (2.0 to 4.9) and (L5S1 (OR = 2.6 (1.7 to 4.0) when comparing the high load to the low load groups.
Luoma et al. 1998	Drivers, carpenters and office workers	Disc bulging	RevMan: Carpenters were more likely to have posterior disc bulging at L3 L4 OR = 2.73; 95% CI 1.12 to 6.64) and anterior bulging at L45 (OR = 2.86; 95% CI 1.05 to 7.79) when compared to the sedentary group. There was no difference for anterior disc bulging at L2 L3 (OR = 2.60 95% CI 0.74 to 9.22), L3 L4 (OR = 3.0 95% CI 0.86 to 10.41) and L5S1 (OR = 1.39 95% CI 0.49 to 3.92) or posterior disc bulging at L2 L3 (OR = 2.53 95% CI 0.60 to 10.69), L4 L5 (OR = 1.92 95% CI 0.89 to 4.16) and L5S1 (OR = 1.33 95% CI 0.95 to 1.87)
Schenk et al. 2006	nurses and office workers	Disc abnormalities (bulging, protusion, extrusion, etc)	RevMan: There was no significant difference in disc bulging between groups. (OR = 1.33 95% CI 0.95 to 1.97)
Videman et al. 2006	Job code (1–4), occupational driving, maximum weight lifted at work (kg)	Disc bulging	There was an association between occupational lifting and disc bulging (0.07 points/disc increase in disc height = 0.065)
Modic changes			
Elfering et al. 2002	Summary score from 0 to 4 combining of lifting or carrying heavy objects, forward bending, vibration and sedentary work	Modic changes (summary score for all levels together)	Occupational loading was not associated Modic changes
Han et al. 201	Work was self-reported and rated as light physical (mainly walking, moderate physical work (sitting/walking) and hard physical work (heavy working)	Modic changes assessed according to Modic et al. and graded into Type I, II or II.	There was a significant difference in the incidence of modic changes according to the level of physical work. In total 8 of 54 (15%) of those with light physical work had modic changes, 16 of 99 (26%) of those with moderate physical load and 23 of 57 (40%) of those with hard physical work had modic changes
Schenk et al. 2006	nurses and office workers	Modic changes	RevMan: There was no difference in Modic changes between groups. (OR = 0.91 95% CI 0.52 to 1.58),
Schmorl’s nodes			
Frymoyer et al. 1984	Lifting	Schmorl’s nodes	There was no association between lifting and the spine outcomes evaluated.
Endplate abnormalities			
Riihimaki et al. 1990	Concrete reinforcement workers and house painters	Endplate sclerosis	RevMan: There was no difference in endplate sclerosis between groups. Overall (OR = 1.97 95% CI 0.96 to 4.05), L1 L2 (OR = 4.70 95% CI 0.22 to 98.43), L2 L3 (OR = 2.84 95% CI 0.57 to 14.25), L3 L4 (OR = 0.93 95% CI 0.13 to 6.66), L4 L5 (OR = 2.84 95% CI 0.57 to 14.25) and L5S1 (OR = 0.83 95% CI 0.33 to 2.09)
Videman et al. 2006	Job code (1–4), occupational driving, maximum weight lifted at work (kg)	Upper endplate irregularities	There was no association between occupational lifting and changes in endplate abnormalities over a 5-year follow-up.
Osteophytes			
Frymoyer et al. 1984	Lifting	Osteophytes	There was no association between lifting and the spine outcomes evaluated.
Riihimaki et al. 1990	Concrete reinforcement workers and house painters	Anterior and posterior spondylophytes	RevMan: Concrete workers had more spondylophytes at L1 L2 (OR = 3.68; 95% CI 1.20 to 11.27), L4 L5 spondylophytes (OR = 3.68; 95% CI 1.20 to 11.27) than painters. There was no difference for overall (OR = 1.68 95% CI 1.05 to 2.69), L2 L3 (OR = 1.84 95% CI 0.84 to 4.06), L3 L4 (OR = 1.97 95% CI 0.96 to 4.05) and L5S1 (OR = 1.43 95% CI 0.63 to 3.25).
Videman et al. 2006	Job code (1–4), occupational driving, maximum weight lifted at work (kg)	Osteophytes	There was no association between occupational lifting and changes in osteophytes over time.
High Intensity Zones			
Videman et al. 2006	Job code (1–4), occupational driving, maximum weight lifted at work (kg)	High intensity zones (signal intensity)	There was no association between occupational lifting and changes in high intensity zones over time.

**Fig. 2:**
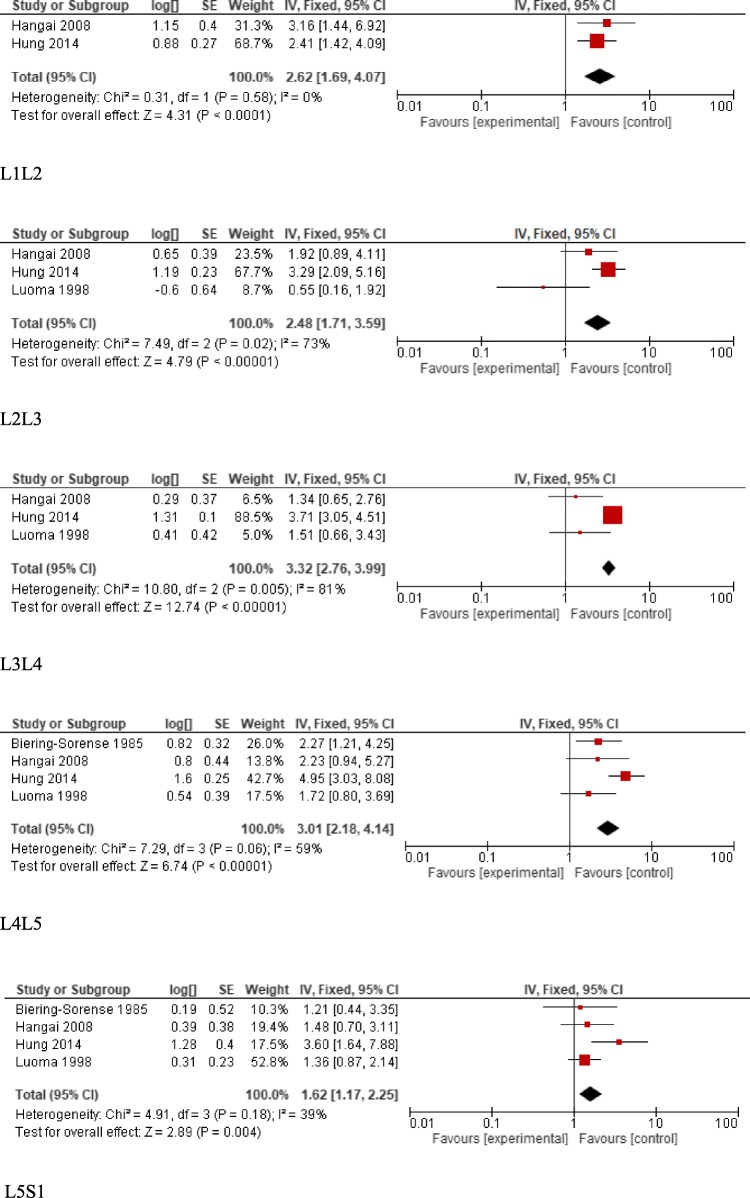
Disc degeneration (signal intensity) forest plots for each spinal level, L1-S1

We were able to pool the results of 4 studies evaluating the association of disc degeneration with different types of occupational load for different spine levels [2–4, 133]. The results demonstrated that for all levels evaluated, including L1-S1, there was a statistically significant difference between loading groups with more degeneration associated with greater loading. Forest plost are presented in Fig. 2.

Seven studies evaluated disc height [2, 125, 127, 128, 131, 133, 137]. Disc height can be measured on imaging using quantitative or qualitative measures and is a surrogate measure of disc degeneration. Of the seven studies evaluating disc height only one did not find a significant difference in disc height between groups [2]. The other six studies identified some type of influence of occupation load with disc height, with greater load being associated with narrower discs. Four studies identified an overall relationship of loading with disc height, without focusing on specific levels [125, 127, 128, 137] and the other studies found different levels to be significantly different [131, 133].

Seven studies evaluated a difference in the prevalence of disc bulge or herniation [3, 126, 128, 130, 133, 136, 137]. Disc bulges or herniations were primarily evaluated through visual observation of images. Of the seven studies, five identified a significant difference between loading groups [3, 128, 133, 136, 137]. Three studies evaluated the prevalence of all lumbar levels together [128, 136, 137] while two studies found difference for different levels, [3, 133] which varied between the studies. When calculated, odd ratios varied between 2.0 to 3.1.

Three studies evaluated Modic changes [126, 129, 135]. Modic changes represent lesions of the vertebral endplate that is adjacent to the bone marrow. Modic changes are often assessed qualitatively [135]. In this review only one study [135] identified a relationship of modic changes with occupation load. One study evaluated the prevalence of Schmorl’s nodes [132]. These are small protrusion of the disc into the vertebral body. The one study included in this review did not identify a relationship of nodes with occupational loading groups. Two studies evaluated the presence of other endplate abnormalities, [125, 137] with only one study identifying a difference between loading groups [137]. Finally, three studies evaluated the presence of osteophytes [125, 132, 137] with two studies identifying greater prevalence of osteophytes in those with greater load [125, 137].

## Discussion

The results of this study suggest that there is moderate grade evidence of an association between occupational loading and disc degeneration in terms of signal intensity. There is low quality grade evidence between loading and disc height, with inconsistent results between levels. There is low quality evidence for an association of disc bulging with occupational loading, again with inconsistent results among spinal levels. There is low quality evidence of an association between occupational loading and osteophytes, Modic changes, Schmorl’s nodes and other endplate abnormalities.

The results do suggest that occupations with greater physical loading are associated with modestly greater spine degeneration although differences in loading conditions and outcomes between studies make it is difficult to draw strong, specific conclusions. This is especially true given that positive results were inconsistently found at different spinal levels and for different outcomes. Thus, it remains difficult to draw conclusions about which type of loading may negatively affect which type of degenerative or structural findings. Additionally, different imaging methods were used (e.g. MRI, CTScans and x-rays) and different methods to assess spine degeneration make it difficult to draw conclusions.

Limitations of the review are primarily related to the heterogeneity of the studies included. There was a wide range of types of occupational loading studied and a wide range of outcomes evaluated. Thus, although odds ratios were presented in the original manuscripts for most of the studies, it was not possible to pool the great majority of results and the findings of the review, therefore, were presented qualitatively. Finally, the poor methodological quality of some of the studies, with only a small portion assessing degeneration longitudinally, limits interpretation regarding the progression of spine degeneration.

Future research should focus on more longitudinal studies, where the development of spinal degeneration can be followed over time, with an adequate follow-up period to allow for structural changes to occur. Monozygotic twin studies should be considered, given the strength of twin study designs in minimizing possible confounding. Furthermore, individual loading exposures should be taken in consideration, especially as the activities and loading involved in any one profession can vary significantly. More specifically, the type and magnitude of loading should be depicted and evaluated in greater detail. Finally, with the advance of imaging techniques and measurement procedures, a wide variety of measures of spinal degeneration and pathology has resulted. Guidelines for measurement and better standardization of spine imaging phenotypes are needed to allow study comparisons and pooling of data to facilitate interpretation of the collective body of related research.

## Conclusion

The results of this study suggest that there is moderate grade evidence of an association between occupational loading and disc degeneration in terms of signal intensity (disc degeneration). There is low or very low-quality grade evidence between loading and disc height, disc bulging, osteophytes, Modic changes, Schmorl’s nodes and other endplate abnormalities. While there seems to be a modest association between heavy occupational loading and spinal degenerative findings, the limitations of the results found in this review provide a weak foundation for practical applications and related health policies.

## Supplementary information

**Additional file 1.** Search strategy section.

## Data Availability

The sources of data used in this study are available within the manuscripts and its supplementary files.

## Abbreviations

CI: Confidence Interval
CTScans: Computerized Tomography scan
GRADE: Grading of Recommendations, Assessment, Development and Evaluations
LBP: Low Back Pain
MRI: Magnetic Resonance Imaging
OR: Odds ratio
r: correlation
