# Supplementary material for: The association between occupational loading and spine degeneration on imaging – a systematic review and meta-analysis
Source: BMC Musculoskelet Disord. 2019 Oct 27;20:489. doi: 10.1186/s12891-019-2835-2 (PMC6815427; doi:10.1186/s12891-019-2835-2)
Supplement: Supplementary file 1 — Additional file 1. Search strategy section. [file 12891_2019_2835_MOESM1_ESM.docx]

# Search terms

MEDLINE

1. intervertebral disc degeneration/ or intervertebral disc displacement/

2. ((spine or spinal or vertebr* or interverteb* or disk* or disc*) adj3 (degenerat* or displace* or injur* or damage* or herniat*)).mp.

3. Lumbago.mp. or exp Low Back Pain/

4. back ache.mp. or exp Back Pain/

5. 1 or 2 or 3 or 4

6. (workload or workplace or work-related or worker* or employee* or job related or occupational or industrial).mp.

7. exp Occupations/

8. exp Physical Exertion/ or Occupation* load.mp.

9. exp "Moving and Lifting Patients"/ or lifting.mp. or exp Lifting/

10. exp Torque/

11. weight bearing.mp. or exp Weight-Bearing/

12. Mechanical stress.mp. or exp Stress, Mechanical/

13. exp Vibration/ or Body vibration*.mp.

14. whole body vibration*.mp.

15. 6 or 7 or 8 or 9 or 10 or 11 or 12 or 13 or 14

16. 5 and 15

17. ra.fs. or imag*.mp. or x-ray*.mp. or us.fs. or ultrasound.mp. or tomograph*.mp. or ct.mp. or scan*.mp. or radiolog*.mp. or radiograph*.mp. or mri.mp.

18. 16 and 17

EMBASE

1. exp intervertebral disk degeneration/

2. ((spine or spinal or vertebr* or interverteb* or disk* or disc*) adj3 (degenerat* or displace* or injur* or damage* or herniat*)).mp.

3. Lumbago.mp. or exp low back pain/

4. back ache.mp. or exp backache/

5. 1 or 2 or 3 or 4

6. exp occupation/ or exp occupational health/ or exp workload/ or exp occupational exposure/ or Occupation* load.mp. or exp occupational disease/

7. exp whole body vibration/ or Vibrations.mp. or exp vibration/

8. lifting.mp. or exp biomechanics/

9. torque.mp. or exp torque/

10. exp mechanical torsion/ or exp torsion/ or torsion.mp.

11. weight bearing.mp. or exp weight bearing/

12. Mechanical stress.mp. or exp mechanical stress/

13. 6 or 7 or 8 or 9 or 10 or 11 or 12

14. 5 and 13

15. (imag* or x-ray* or radiolog* or radiograph* or ultrasound or tomograph* or ct or mri or scan*).mp. [mp=title, abstract, subject headings, heading word, drug trade name, original title, device manufacturer, drug manufacturer, device trade name, keyword]

16. 14 and 15

CINAHL until October 2015

S1 "Occupation" OR "Occupation*" Or "Occupation* Load" OR "Lifting" OR "Torque" Or "weight bearing" Or "Mechanical Stress" Or "vibration*" Or "whole body vibration"

S2 ( (spine or spinal or vertebr* or interverteb* or disk* or disc*) n3 (degenerat* or displace* or injur* or damage* or herniat*) ) OR (MH "Intervertebral Disk Displacement")

S3 embase

S4 (S1 and S2 and S3)

CINAHL EBSCO March 13 2017

1. (MH "Occupation (Human)") OR "occupation" OR (MH "Occupations and Professions")
2. (MH "Occupational Diseases") OR (MH "Injury, Occupational Disease, Poisoning")
3. (MH "Occupational Exposure")
4. (MH "Occupational Hazards")
5. (MH "Occupational Health")
6. "occupational load"
7. (MH "Lifting") OR "lifting" OR (MH "Lifting and Transfer Equipment") OR (MH "Weight Lifting")
8. (MH "Torque") OR "torque"
9. (MH "Weight-Bearing") OR "weigth bearing"
10. (MH "Stress, Mechanical") OR "mechanical stress"
11. (MH "Vibration") OR "vibration"
12. "whole body vibration"
13. (MH "Spine") OR "spine"
14. "spinal"
15. "spine degeneration"
16. (MH "Intervertebral Disk")
17. "disc degeneration"
18. "degenerative disk disease"
19. (MH "Lumbar Vertebrae") OR "lumbar vertebrae"
20. S1 OR S2 OR S3 OR S4 OR S5 OR S6 OR S7 OR S8 OR S9 OR S10 OR S11 OR S12
21. S13 OR S14 OR S15 OR S16 OR S18 OR S19
22. S20 AND S21
